# Glutamate Pays Its Own Way in Astrocytes

**DOI:** 10.3389/fendo.2013.00191

**Published:** 2013-12-16

**Authors:** Mary C. McKenna

**Affiliations:** ^1^Department of Pediatrics and Program in Neuroscience, University of Maryland School of Medicine, Baltimore, MD, USA

**Keywords:** glutamate, astrocytes, oxidative metabolism, glutamate dehydrogenase, pyruvate recycling pathway

## Abstract

*In vitro* and *in vivo* studies have shown that glutamate can be oxidized for energy by brain astrocytes. The ability to harvest the energy from glutamate provides astrocytes with a mechanism to offset the high ATP cost of the uptake of glutamate from the synaptic cleft. This brief review focuses on oxidative metabolism of glutamate by astrocytes, the specific pathways involved in the complete oxidation of glutamate and the energy provided by each reaction.

## Introduction

One of the most essential roles of astrocytes in brain is removal of the neurotransmitter glutamate from the synaptic cleft as it is crucial that a low resting glutamate concentration of ∼1–10 μM be maintained for continued glutamatergic neurotransmission and brain function (1–3). Astrocytes perform this key function by rapidly and efficiently removing glutamate which increases orders of magnitude in concentration to ∼100 μM–1 mM after depolarization of neurons (4, 5). Uptake of glutamate is a very expensive proposition since the astrocyte transporters that mediate glutamate transport 3 Na^+^ molecules which must be exported by the enzyme Na^+^, K^+^-ATPase. Thus the uptake of one molecule of glutamate by an astrocyte requires the expenditure of one molecule of ATP (1). To pay the high cost of removing large amounts of glutamate from glutamatergic synapses, astrocytes must form large amounts of ATP from the metabolism of glucose or other substrates (see Figure 1A; Table 1). About 30% of the oxidative metabolism in brain *in vivo* takes place in astrocytes (6–9); however, it is not likely that these cells oxidize sufficient glucose to generate the ATP required for the transport of such massive amounts of glutamate (10–16). A number of groups have shown that astrocytes have a sufficiently high rate of glutamate oxidative metabolism to pay the high cost of glutamate uptake (11, 17, 18). This short review summarizes the information on the use of glutamate in astrocytes and recent evidence on the role of protein complexes in facilitating glutamate metabolism (19, 20). The goal of this paper is to provide a short, very concise, and focused review, and to point readers to many excellent more in depth manuscripts recently published (2, 3, 6, 16, 21).

**Figure 1A:**
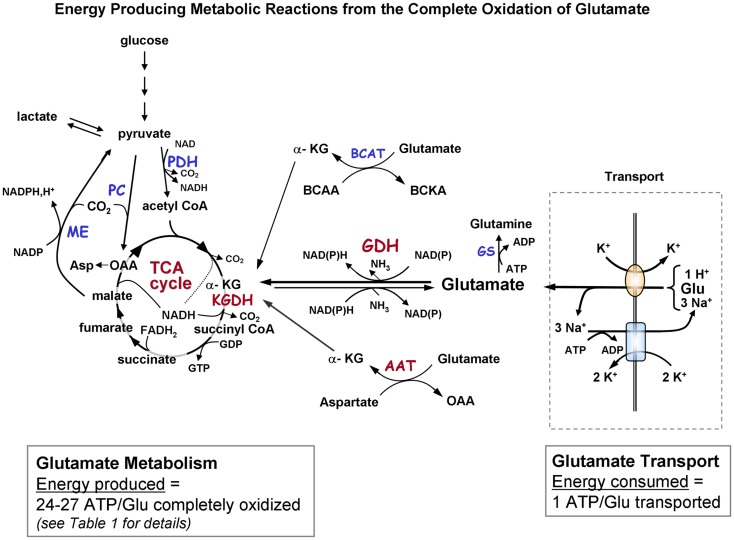
**Schematic diagram indicating the pathways of metabolism of *exogenous* glutamate taken up by astrocytes**. The portion of the figure outlined with a dashed line shows transport of glutamate from the extracellular milieu. The oval depicts the astrocytic glutamate transporter GLT1/EAAT2 or GLAST; rectangle depicts Na^+^, K^+^-ATPase. Astrocytic glutamate can be converted to α-ketoglutarate by the energy producing enzyme glutamate dehydrogenase or via one of the transaminase enzymes (primarily via aspartate aminotransferase but also by BCAT and ALAT). α-Ketoglutarate formed from glutamate is metabolized via a partial TCA cycle to either malate or oxaloacetate. For the *complete oxidation* of glutamate to occur malate (or OAA) must leave the TCA cycle, be converted to pyruvate by malic enzyme, and then converted to acetyl CoA by pyruvate dehydrogenase. The acetyl CoA formed by these pyruvate recycling reactions re-enters the TCA cycle and is subsequently oxidized for energy. The dotted in the TCA cycle indicates that the NADH generated from the isocitrate dehydrogenase reaction would only be formed when the acetyl CoA formed from the pyruvate recycling pathway is metabolized via the TCA cycle because the initial entry of carbons α-KG at is subsequent to this step. The complete oxidation of one glutamate molecule can yield 24–27 ATP depending on whether the initial step in metabolism is via GDH or AAT. The ATP is estimated at ∼20 since the maximum theoretical yield of ATP is never recovered due to mitochondrial proton leak. [See Table 1 for reaction details and **(B)** for labeling pattern of metabolites labeled from the initial metabolism of [U-^13^C]glutamate and the labeling pattern from subsequent metabolism via the pyruvate recycling pathway and re-entry into the TCA cycle]. Abbreviations: Glu, glutamate; α-KG, α-ketoglutarate OAA, oxaloacetate; Asp, aspartate; GDH, glutamate dehydrogenase; AAT aspartate aminotransferase; BCAT, branched-chain aminotransferase; ALAT, alanine aminotransferase; KGDH, α-ketoglutarate dehydrogenase; ME, malic enzyme; PDH, pyruvate dehydrogenase; GS, glutamine synthetase. BCAA, branched chain amino acid; BCKA, branched chain ketoacid.

**Figure 1B:**
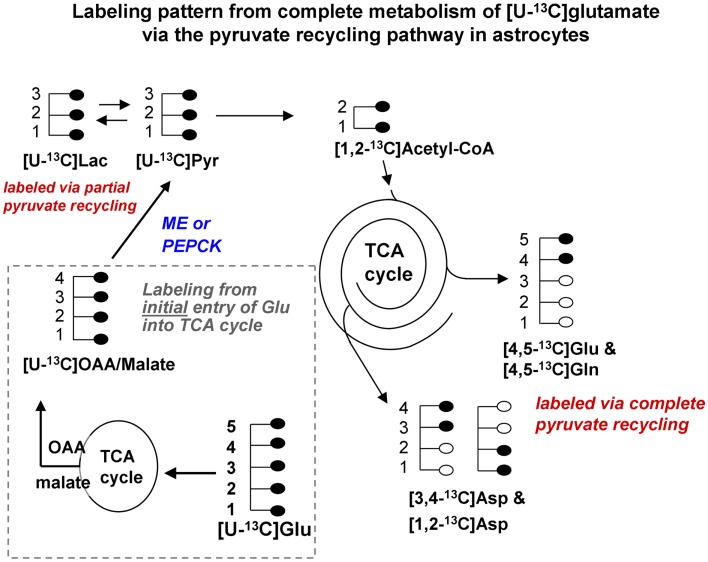
**Labeling pattern from the initial metabolism of [U-^13^C]glutamate and the labeling pattern from the complete oxidation of these glutamate carbons via the pyruvate recycling pathway and re-entry into the TCA cycle**. Labeling from the initial entry of [U-^13^C]glutamate into the TCA cycle is shown inside the dotted line. Note that metabolites generated are also uniformly labeled in all carbons. [Note that any glutamine formed in the cytosol directly from [U-^13^C]glutamate would also be uniformly labeled [U-^13^C]glutamine; *reaction not shown in this figure*]. [U-^13^C]malate or OAA leaving the TCA cycle would be converted to [U-^13^C]pyruvate by malic enzyme or the combined action of PEPCK and pyruvate kinase, and would also give rise to [U-^13^C]lactate. The [U-^13^C]pyruvate can be converted to [1,2-^13^C]acetyl CoA which enters the TCA cycle for further oxidation. Any citrate formed from the condensation of the [1,2-^13^C]acetyl CoA with *unlabeled* oxaloacetate in the cycle would give rise to [4,5-^13^C]glutamate and glutamine, and also to both [1,2-^13^C]aspartate and [3,4-^13^C]aspartate. These partially labeled glutamate, glutamine, and aspartate molecules can be readily distinguished from the [U-^13^C]glutamate, glutamine, and aspartate by ^13^C-NMR spectroscopy. It is very likely that studies of pyruvate recycling from [U-^13^C]glutamate underestimate the amount of recycling taking place since any citrate formed from the condensation of [1,2-^13^C]acetyl CoA with [U-^13^C]OAA formed from the initial entry and metabolism of the glutamate into the TCA cycle give rise to [U-^13^C]glutamate which can not be distinguished from the precursor. Abbreviations: Glu, glutamate; Gln, glutamine; OAA, oxaloacetate; Asp, aspartate; Lac, lactate; Pyr, pyruvate; ME, malic enzyme; PEPCK, phosphoenolpyruvate carboxykinase.

**Table 1:** **Energy produced in astrocytes from oxidation of one glutamate molecule in the TCA cycle and oxidation via the pyruvate recycling pathway**.

**Energy required for uptake of one molecule of glutamate**			
**Uptake by EAAT2 (GLT1) or GLAST**		**Rebalancing ions**		**ATP used**
1 Glutamate + 3 Na^+^ + 2 K^+^ + 1 H^+^ taken up	→	3 Na^+^ extruded		1
**Energy provided by complete oxidation of one molecule of glutamate**
**Reaction**			**ATP equivalents (theoretical maximum)**	
**GDH (glutamate dehydrogenase) reaction**
Glutamate → α-ketoglutarate			NADPH	3
**ATP equivalents from one Glu metabolized via GDH rxn**				**3**
*or* ATP equivalents from AAT rxn				0
**TCA cycle reactions**
α-Ketoglutarate → succinyl CoA			NADH	3
Succinyl CoA → succinate			GTP	1
Succinate → fumarate			FADH2	2
Fumarate → malate				0
**ATP equivalents produced from one Glu metabolized via partial TCA cycle to malate**				**6**
**Malate can stay in the TCA cycle or be metabolized via pyruvate recycling pathway:**	
(calculations below assume that carbons from one Glu is metabolized via pyruvate recycling)	
Carbons from one Glu staying in TCA cycle (rather than pyruvate recycling)				
Malate → oxaloacetate			NADH	3**
**Carbons from one Glu metabolized via the pyruvate recycling pathway**
**Pyruvate recycling pathway**
Malate → pyruvate			NADPH	3
Pyruvate → acetyl CoA			NADH	3
*From acetyl moiety derived from one glutamate re-entering TCA cycle*
Acetyl CoA → oxaloacetate (one complete turn of TCA cycle)			3 NADH	9
			1 FADH_2_	2
			1 GTP	1
**ATP equivalents from pyruvate recycling and oxidation of acetyl moiety**				18**
**Total ATP from complete oxidation of one molecule of exogenous glutamate**
**via the TCA cycle and pyruvate recycling pathway**				24–27*
**NET energy yield from uptake and oxidation of one glutamate molecules**				23–26***

*Values with asterisks includes the ATP generated from the reoxidation of NADPH formed during conversion of glutamate to α-ketoglutarate in the reaction catalyzed by glutamate dehydrogenase (GDH).

**Note that when the carbon skeleton from metabolism of glutamate leaves the TCA cycle as malate to proceed through the pyruvate recycling pathway, then NADH will not be formed by malate dehydrogenase (MDH) which converts malate → OAA. However, with multiple molecules of glutamate entering oxidative metabolic pathways in astrocytes some of the glutamate would be converted to OAA and be used for formation of citrate and producing NADH at the MDH step. Note that if a glutamate molecule stays in the TCA cycle 9–12 molecules of ATP would be produced which is less than when it is metabolized via the pyruvate recycling pathway but still considerably more than the ATP required for glutamate transport.

***The total ATP generated would be 27 if glutamate → α-ketoglutarate proceeds via the GDH reaction, and only 24 if it proceeds via AAT. The ATP values are estimates as noted in Figure 1A since the maximum theoretical yield of ATP is never recovered due to the mitochondrial proton leak.

## What is the Evidence that Glutamate is Metabolized by Astrocytes?

It is well established that astrocytes can oxidize glucose and other substrates for energy including lactate, glutamate, glutamine, fatty acids, and the ketone bodies 3-hydroxybutyrate and acetoacetate (12, 17, 22–30). These substrate are actively oxidized for energy; however, glutamate is oxidized by astrocytes at a rate much higher than the other substrates. The oxidation of glutamate by astrocytes was initially determined with studies using radiolabeled ^14^C-glutamate (12, 30–32). However, the more recent use of ^13^C-glutamate and ^13^C-NMR spectroscopy has provided more complete information about the metabolic fate of glutamate in astrocytes. Sonnewald et al. (18) first reported that more of the label from glutamate metabolism was incorporated into lactate by astrocytes than was converted to glutamine. This key finding was initially considered controversial as it underscored that the glutamate-glutamine cycle is not stoichiometric since only a portion of the glutamate taken up by astrocytes was converted to glutamine. A key study by the McKenna and Sonnewald (29) groups demonstrated that when the exogenous glutamate concentration was increased from 0.1 to 0.5 mM the proportion of glutamate oxidized by the TCA cycle in astrocytes greatly increased and the percent converted to glutamine decreased. Reports from many groups clearly demonstrate (17, 29, 30, 33) that astrocytes have the capability to oxidize the concentrations of glutamate present in the synaptic cleft after depolarization of neurons (100 μM–1 mM) (4). Hertz and Hertz (17) noted that glutamate oxidation by astrocytes is as high as the anaplerotic rate of glutamate production suggesting that synthesis must be balanced by catabolism as glutamate does not readily exit the brain. A recent report by our group (11) showed that glutamate was oxidized by astrocytes at a rate higher than glucose, 3-hydroxybutyrate, glutamine, lactate, or malate, and that none of the other substrates could effectively decrease the oxidative metabolism of glutamate.

Data from several different types of studies provide evidence that suggests or demonstrates that glutamate oxidation occurs in astrocytes *in vivo*. These include *in vivo* microdialysis studies demonstrating oxidation of glutamate in the hippocampus of freely moving rats (34, 35), evidence from several groups documenting that the fine processes of astrocytes enveloping synaptic terminals contain abundant mitochondria (6) (and Tibor Kristian, unpublished), and transcriptome studies on astrocytes isolated from brain of adult rodents that document very high levels of transcripts for glutamate dehydrogenase (GDH) and for enzymes of the TCA cycle (6).

## Oxidation of the Carbon Skeleton of Glutamate Offsets the Cost of Glutamate Uptake

Glutamate taken up by astrocytes can be converted to α-ketoglutarate by two reactions, either by transamination reactions or by the energy producing reaction of the enzyme GDH which is enriched in astrocytes (3, 6, 36, 37). Transamination occurs primarily by aspartate aminotransferase (AAT), but also readily takes place via either branched-chain amino acid aminotransferase (BCAT) or alanine aminotransferase (ALAT) (3, 38–40). Studies from our group and others demonstrate that the oxidative metabolism of *exogenous* glutamate taken up from the extracellular milieu proceeds *primarily* via GDH in astrocytes from rat brain [since it is relatively unaffected by the transaminase inhibitor aminooxyacetic acid, AOAA] (30, 41). The α-ketoglutarate formed from glutamate is metabolized for energy in the sequential reactions of the TCA cycle to the four carbon compound oxaloacetate (Figures 1A,B) and yielding the equivalent of nine ATP molecules in this process.

## The Complete Oxidation of Glutamate Requires Metabolism of Part of the Carbon Skeleton via the Pyruvate Recycling Pathway

Studies using ^13^C-NMR spectroscopy have provided key insights into the metabolic fate of glutamate in astrocytes and information about the compartmentation of glutamate metabolism (28, 29, 42–44). Several groups have shown that the carbon skeleton of glutamate can enter the TCA cycle leading to labeling in aspartate and lactate (28, 29, 42–44). The incorporation of label from [U-^13^C]glutamate into [U-^13^C]lactate (see Figure 1B) and also into [1,2-^13^C]glutamate and glutamine and specifically labeled molecules of aspartate confirms that the carbon skeleton of glutamate can be metabolized via the pyruvate recycling pathway in astrocytes and reenter the TCA cycle. Thus, all carbons of the glutamate molecule can be completely oxidized for energy via the TCA cycle and pyruvate recycling pathway (45) (see Figure 1B).

## Other Substrates Can Facilitate the Uptake and Oxidative Metabolism of Glutamate by Astrocytes

The high rate of glutamate oxidation reported by several groups is consistent with the earlier findings of Hertz and Hertz (17) demonstrating that 100 μM glutamate supported O_2_ uptake by astrocytes as effectively as 7.5 mM glucose. Hertz also demonstrated that O_2_ uptake and respiration was significantly higher with the combination of glutamate + glucose than with either substrate alone (17). Data from several studies suggests that glucose may facilitate the uptake and oxidation of glutamate by astrocytes. McKenna et al. (30) showed that the presence of 1 mM pyruvate increased the rate glutamate oxidation by astrocytes, possibly by increasing transamination to α-ketoglutarate and metabolism via the TCA cycle in the presence of pyruvate. However, we did not find any effect of glucose or lactate on rate of ^14^CO_2_ production from [U-^14^C]glutamate in a recent study (11). In contrast, studies by some groups showed that glutamate uptake stimulated glycolysis in astrocytes; however, this has not been found by all groups (46, 47).

## Oxidation of Glutamate for Energy Spares Glucose and Other Substrates

Substrate competition studies recently reported by our group demonstrated the robustness of glutamate use by astrocytes as none of the other substrates added, including glucose, had the ability to decrease the oxidation of glutamate (11). Hertz and Hertz (17) found higher respiration and O_2_ consumption when astrocytes were incubated in the presence of glutamate plus glucose and that the addition of glutamate spared glucose consumption. Earlier studies by Peng et al. (48) showed that added glutamate decreased the rate of glucose oxidation in astrocytes by 75%.

## Astrocyte Glutamate Transporters and Mitochondrial Proteins Form Complexes That Facilitate Oxidation of Glutamate for Energy in Astrocytes

Recent reports from the Robinson lab (19, 20) demonstrate that glutamate uptake by astrocytes is tightly associated with a multi protein complex which includes the glial glutamate transporters, hexokinase and mitochondrial proteins suggesting that there is a mechanism in astrocytes that insures that a portion of the glutamate taken up is selectively delivered to mitochondria for oxidative energy metabolism. They demonstrated that the astrocyte glutamate transporter GLT1 can co-compartmentalize with hexokinase, other glycolytic enzymes, GDH, and mitochondria (20, 49). A report from the Robinson group in this special issue (49) suggesting that the enzyme GDH associates with the astrocyte glutamate transporters strengthens the evidence for the formation of a protein complex to facilitate the mitochondrial oxidation of glutamate.

Overall, there is compelling data from both *in vitro* and *in vivo* studies that oxidative metabolism of glutamate occurs in astrocytes and provides sufficient energy to pay for the cost of glutamate uptake from the synaptic cleft.

## Conflict of Interest Statement

The author declares that the research was conducted in the absence of any commercial or financial relationships that could be construed as a potential conflict of interest.
